# Recent Advances in Cellulose Chemistry

**DOI:** 10.3390/ijms25168977

**Published:** 2024-08-18

**Authors:** Salah-Eddine Stiriba

**Affiliations:** Instituto de Ciencia Molecular/ICMol, Universidad de Valencia, C/Catedrático José Beltrán 2, 46980 Valencia, Spain; stiriba@uv.es

The current transition from fossil-based raw materials to biomass-based materials, and their application, has led to the innovation and development of sustainable chemical industries towards a circular economy. Naturally occurring polymers, namely, biopolymers, such as the polysaccharide cellulose, present a renewable and abundant raw material, with fascinating structures and properties [1]. Cellulose constitutes the best example of the merging of carbohydrate and polymer chemistry. Consequently, the biocompatible, biodegradable cellulose macromolecule, and its derivatives, are increasingly being utilized as innovative materials in a wide range of domains and technologies, including in coatings, films, membranes, building materials, and pharmaceuticals [2,3,4].

This Special Issue of *International Journal of Molecular Science* aims to follow the recent advances made in cellulose chemistry in both academia and industry. It features six contributions, five of which are research articles and the sixth a review article.

In its first contribution, H. Huang and co-workers (DOI: 10.3390/ijms241914431) developed size-controlled silver nanoparticles supported by pyrolytic carbon prepared from microcrystalline cellulose. This newly prepared functional cellulose nanocomposite demonstrated high electric conductivity and strong antimicrobial activity against *Escherichia* coli bacterium.

In the second article, L. Bahsis (DOI: 10.3390/ijms24119301) illustrates the power of cellulose as abio-support for catalyst in organic chemistry. In fact, a copper catalyst was supported on the polysaccharide cellulose acetate and used as a heterogenous catalyst in the copper-catalyzed azide-alkyne [3+2] cycloaddition click reaction (CuAAC). The design of this heterogenous catalyst and its recovery and recycling constitute an advance in the sustainability of the CuAAC within the click chemistry concept.

A new frontier in cellulose biomaterials generated by some aerobic bacteria has also been addressed in this Special Issue. A. Vassil’kov and co-workers (DOI: 10.3390/ijms24087667) reported the synthesis and characterization of new Ag nanoparticle-containing nanocomposites obtained by modifying various forms of bacterial cellulose with Ag nanoparticles prepared *via* metal–vapor synthesis. Such new Ag-containing composites exhibit antimicrobial activity against *Bacillus subtilis*, *Staphylococcus aureus*, and *Escherichia coli* bacteria, as well as *Candida albicans* and *Aspergillus niger* fungi.

Meanwhile, C. Zhang (DOI: 10.3390/ijms24055036) report a facile and effective strategy for the fabrication of mechanically enhanced thermoplastic polyurethane composite films with excellent mechanical properties using modified cellulose nanocrystals.

Ogiwara, Iwata, and Furumi (DOI: 10.3390/ijms24054269) investigate a promising synthetic eco-friendly strategy for the fabrication of thermotropic cholesteric liquid crystals with visible reflection, using hydroxypropyl cellulose and derivatives bearing alkanoyl side chains. 

This Special Issue of *IJMS* ends with a review article by J. C. Parajó and co-workers (DOI: 10.3390/ijms241512404) on the state-of-the-art technology of cellulose manufacturing in biphasic reaction media. In their article, they describe the fundamentals of industrial cellulose pulp production processes using organosolvent methods.

Some of the recent advances in the field of cellulose research and applications have been addressed through these contributions. Cellulose, a fantastic and exhaustive natural polymer, holds significant potential for expanding fundamental knowledge in this field, as well as for application at an industrial scale.

## References

[1] Klemm D., Heublein B., Fink H.-P. (2005). Bohn, Cellulose: Fascinating Biopolymer and Sustainable Raw Material. Angew. Chem. Int. Ed..

[2] Lukova P., Katsarov P., Pilicheva B. (2023). Application of Starch, Cellulose, and Their Derivatives in the Development of Microparticle Drug-Delivery Systems. Polymers.

[3] Ahn E., Kim T., Jeon Y., Kim B.-S. (2020). A4 Paper Chemistry: Synthesis of a Versatile and Chemically Modifiable Cellulose Membrane. ACS Nano.

[4] Roy S., Zhai L., Kim J.W., Kim H.C., Kim J. (2020). A Novel Approach of Developing Sustainable Cellulose Coating for Self-Cleaning-Healing Fabric. Prog. Org. Coat..

